# The Value of a Motor Intervention for 3 to 6-Year-Old Children Infected with and Affected by HIV

**DOI:** 10.3390/ijerph19052967

**Published:** 2022-03-03

**Authors:** Anita Elizabeth Pienaar, Jo-Anne Elizabeth Botha

**Affiliations:** Physical Activity, Sport and Recreation Focus Area, (PhASRec) Faculty of Health Science, North-West University, Potchefstroom 2520, South Africa; joanne@yrless.co.za

**Keywords:** HIV, children, motor milestones, motor skills, motor intervention, neurodevelopmental delays, psychomotor

## Abstract

Human immunodeficiency virus (HIV)/Aquired Immune Deficiency Syndrome (AIDS) is a large threat to human health and is challenging to address. This study aims to determine if motor intervention is a possibility for promoting the life expectancy and quality of life of children with HIV. The group consisted of 22 participants: 11 HIV-infected (51.73 months, SD 10.15) and 11 HIV-affected children (44.45 months, SD 10.76). A two-group (intervention and control group) pre-test–post-test research design was followed. The HIV-infected and affected children were randomly matched and grouped into an intervention and control group. The intervention group participated in a 12-week motor intervention of 60 min per session, twice per week. The effect of the program was analyzed with regard to motor skills, as established by the PDMS-2 and two strength capabilities. An ANCOVA adjusted for pre-test differences (*p* < 0.05) indicated statistically significant improvement (*p* < 0.05) with large practical significance (d > 0.8) in locomotor, fine motor and overall motor skills. The infected children also showed better improvement compared to the affected children. Motor intervention is recommended in the health care path of children affected and infected with HIV, although modifications for improvement of the program are suggested, based on the results attained.

## 1. Introduction

The human immunodeficiency virus (HIV)/Acquired Immune Deficiency Syndrome (AIDS) has become one of the largest threats to human health over the past two decades [1,2], and therefore is one of the largest challenges to address [3,4]. The influence of HIV/AIDS is complex; consequently, it involves many implications for planning health services [5]. Globally, 37.7 million people lives with HIV of which 20.6 million lives in Eastern and Southern Africa. In the age group 0–14 years, 75,000 children were newly infected in Eastern and Southern Africa in 2020 [6]. 

Research show that children with HIV display neurodevelopmental, cognitive, motor and nutritional deficits [7,8,9]. A comprehensive study at six sub-Saharan Africa study sites in four countries in Africa provides conclusive evidence that African children with HIV are at significant neuropsychological risk, even with early ART treatment initiation and careful medical support [10]. Researchers [4,9,11,12,13] all report motor deficits in HIV-positive children from a young age. Findings [11] in this regard show that children who are HIV positive already display deficits in developmental milestones in the first two years of their lives, while it was found that these deficits already appear during the first three months of a baby’s life and that they gradually intensify as the child needs to be able to execute more complex and integrated tasks [8,11]. Statistically significant differences in cognitive and motor development in HIV-infected children compared to healthy children are also reported in a study at 18 months [4] where 72% had severe motor delay at the baseline measurements of the study. Findings of studies also indicate that delays in motor development, especially gross motor skills, most strongly differentiate HIV infected from HIV-exposed children, and are seen more frequently in infants than school-aged children [2]. A meta-analysis [13] reported that rates of severe motor delays determined by using the BSID varied but ranged from 14% to 81%, and in most of the studied papers researchers reported rates of severe motor delay between 21% and 66%. Children demonstrating motor dysfunction, including abnormal muscle tone, less muscle bulk or less muscle strength, have been shown to be at an increased risk for disease progression [14]. Executive functions such as slowed information processing are also reported to be compromised in HIV children [15]. HIV is furthermore associated with exhaustion and a decline in physical functioning along with other capabilities, which restrict the child from performing life-sustaining activities [16]. The ability to attend school is influenced in up to 50 percent of children, again comprising success in school [16]. Consequently, these children will also not be capable to participate in age appropriate activities such as physical games and sport, or to perform self-care activities such as bathing themselves, if specific attention is not given to the development of gross and fine motor skills and muscle strengthening. Researchers indicate that it is therefore essential for children with HIV to receive motor intervention, since it can promote activities of daily living, life expectancy as well as their quality of life [4,17,18,19,20]. Maintenance of motor skills in the aforementioned children can further enable them to participate in activities which directly contribute to cognitive growth, social skills, perceptual motor skills activities of daily living and executive functioning skills that can again improve school readiness [4,20,21,22,23]. 

Although achieving viral suppression on antiretroviral therapy (ART) results in significant improvements in markers of neurodevelopmental function of young HIV-infected children, potential neurodevelopmental delays still persisted in a large proportion [24]. Despite ART intervention, some children still demonstrated significant, persistent delays in cognitive, language and motor development in another study [25]. Further interventions are therefore needed to limit potential disabilities and maximize developmental outcomes [24]. In this regard, it is contended [26] that the complexity and multiplicity of the problems of HIV+ children require multidisciplinary interventions based on the child as a whole. In agreement, a review [27] suggested that behavioral interventions are needed in combination with medical treatment and care in order to fully address the neurodevelopmental needs of children and adolescents in Africa living with HIV. Such combination ARV/behavioral interventions for children with HIV in resource-constrained settings should also be initiated as early as possible in children’s development, as younger HIV+ children also have the greatest potential to benefit from early interventions in the hopes of preventing further neuropsychological injury from pediatric HIV disease [27,28]. 

Identification of developmental delays in children before the age of three years, and the subsequent implementation of early intervention programs, has the potential to improve long-term developmental outcomes [29,30]. It is also highlighted [19] that an improvement in lifestyle can also bring about an increasing number of HIV positive children surviving to adolescence and some to an even more advanced age, and many with relatively good health. Researchers [4,20,31] are therefore of the opinion that it is important to develop and evaluate complementary health-promoting interventions such as early child development and activity programs that can stimulate growth and development and social maturation at a young age as potential contributors to promote quality of life, since HIV is increasingly becoming a chronic condition. From the literature, it became clear that although the necessity of health-promoting intervention studies performed on children who are infected with and affected by HIV/AIDS are accentuated, limited efforts are reported where developmental interventions that focused on motor intervention have been undertaken on these children as yet [4]. This study is therefore aimed at investigating the value of a motor intervention for 3- to 6-year-old children infected with and affected by HIV as a possible health promotion treatment, and to compare the effects of the intervention on the infected and affected subjects who participated in the intervention.

## 2. Materials and Methods

### 2.1. Investigating Group and Procedures

**Figure 1:**
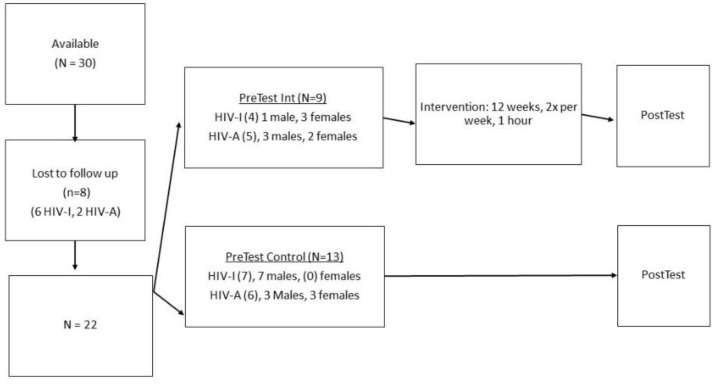
Flow diagram of research design.

A two-group (intervention group and control group) pre-test–post-test-research design were followed where the intervention group was exposed to an intervention program for 12 weeks of 60 min duration, two times per week (see the flow diagram in Figure 1 that displays the research design. 

Following the pre-test and before the onset of the intervention, the HIV-infected and HIV-affected children were divided into an intervention and control group by means of random matching. This grouping was performed because the intention was to establish the effect of the intervention on children that are affected differently by HIV. Thirty children were initially available for the study but eight were lost-to-follow-up (six HIV infected, two HIV affected), mainly as a result of changing circumstances at home or relocation. The group size of the intervention and control groups as well as the number of infected and affected children consequently also differed. The 22 participants that completed the study, had a mean age of 49.64 months, SD 11.08 and comprised of 9 children in the intervention group and 13 in the control group, with both groups including matched infected and affected subjects. The intervention group of nine children included four boys, five girls, mean age 24.55 months, SD 9.13), of which four were HIV-infected children (one boy, three girls) and five HIV-affected (three boys, two girls). The control group comprised 13 children with a mean age of 49.84 months, SD 11.96 (10 boys and 3 girls) of which 7 were HIV-infected (7 boys, 0 girls) and 6 HIV-affected (3 boys, 3 girls) (see Table 1 and Figure 1 which reflects the research design). Both groups included children in the age range between 3 and 6 years, although the majority of the group were in the age range between 3 and 4.5 years. The ages within the various groups as well as genders differed slightly because we had lost participants to follow-up and since this was an availability sample. For ethical reasons, the control group was also exposed to an intervention program after completion of the research.

The HIV-infected group and HIV-affected groups were selected for the study from a Hospice daycare center for HIV-infected and HIV-affected children in Potchefstroom (South Africa). Children are only allowed entrance to this daycare facility if proof of their HIV status can be provided, while medical clinics also refer HIV-positive children to this facility. The affected children (HIV-negative status), are allowed access to the daycare center solely on the proviso that a death certificate of one of or both the parents is provided which states that the death was as a result of an AIDS-related disease such as tuberculosis, pneumonia or cardiac failure. The children from both these groups were transported to and from the school with a school bus on a daily basis.

The HIV status of the children was determined using the FIRST RESPONSE HIV CARD TEST 1-2.O. The test is an immunochromatographic (rapid) test for the qualitative detection of all isotypes (IgG, IgM, IgA) specific to HIV-1 including subtype O and HIV-1 in human serum, plasma or whole blood. In a comparison of the FIRST RESPONSE HIV CARD TEST 1-2.O test versus a leading commercial anti-HIV1and2 ELISA and Rapid test, results gave a sensitivity of 100% (120/120), a specificity of 99.18% (121/122) and a total agreement of 99.59% (241/242). Due to only three laboratories processing PCR testing in South Africa—22% of the total capacity required—rapid tests were used (Meyers et al., 2006). 

The socioeconomic circumstances of the group were considered low because their living conditions were characterized by poor sanitary conditions and housing. Although the diet was not compiled by a dietician, they received food from the school daily. This consisted of maize porridge, morvite or soya porridge for breakfast and a fruit for a snack during the course of the morning. Cooked lunches consisted of meat, rice and vegetables with a peanut butter or jam sandwich and at 15:00, a cold drink before going home. The children were also supplied with morvite over the weekends when the school was closed. 

### 2.2. Ethichal Considerations

The North-West University in Potchefstroom, South Africa provided ethical approval to conduct the study (nr. 06M02). Having received permission from the director of the Hospice in question and from the completed informed consent forms of the parents/legal guardians, the child was included in the study. Each child also had to provide oral assent to be part of the study. The HIV status of the child was determined by the clinic responsible for their health as reported above. 

### 2.3. Measurement Instruments and Apparatus

Peabody Developmental Motor Scales—second edition (PDMS-2).

The PDMS-2, compiled by Folio and Fewell (2000) [32], consists of six subtests, which measure interdependent abilities during early motor development. It was developed to measure gross and fine motor skills in children from birth to 71 months of age. The subtests consist of reflexes, stationary, locomotor skills, object manipulation skills, grasping and visual motor integration. The totals of the subtests are presented as a raw score, a percentile, age equivalents, as well as a standard score. The grading of motor development is represented as follows: (1) Very poor; (2) Poor; (3) Below average; (4) Average; (5) Above average; (6) Excellent and (7) Superior. It is indicated [32] that the standard score gives the best indication of an individual’s progress in the subtests; therefore it is recommended to be used to compare the subtests with one another. These subtests contribute to a gross motor total (reflex, stationary, locomotor skills and object manipulation skills), a fine motor total (grasping and visual motor integration) and an overall motor total. Reflexes are only measured from birth to 12 months; consequently they were not used in this study. The gross motor total and the fine motor total as well as the total motor total are expressed in percentile as well as quotients. The aforementioned is therefore seen as the most reliable value for PDMS-2 [32], because these quotients integrate the various subtests and are not reliant on a single subtest and display the child’s abilities with regard to gross motor, fine motor as well as total motor abilities. The PDMS-2 has been tested by Folio and Fewell (2000) as a reliable and valid measuring instrument. The test–retest-reliability coefficient is >0.90, while the internal validity varies between 0.90 and 0.96. The content validity of the PDMS-2 is determined by the skills which are measured and is corroborated by knowledge of motor development which is already available. It was also found that the test battery was suitable for use with any race, sex or ethnicity.

Physical fitness:

The following strength tests were additionally added to test physical fitness:

Left and right handgrip strength (kg), measured using the Lafayette handgrip dynamometer [33].

Standing long-jump—the standing long-jump action was firstly demonstrated to the participant by the tester.The participant had to stand with the feet next to each other with the toes touching the starting line (0 cm). The participant then had to bend the knees and then jump forward as far as possible. The distance jumped was taken at the back of the heels after landing, unless a step back was taken or a fall occurred; then the measurement was taken from the body part closest to the starting line. Each participant was afforded two opportunities and the best result was noted. Trained translators were used to ensure that the children understood the instructions.

### 2.4. Intervention

The intervention took place at the daycare centre during school hours and was conducted by a registered pediatric exercise scientist called a Kinderkinetict. In this profession, Kinderkineticists are trained to design, adapt and apply motor development programs to children from very young ages, therefore the researcher was familiar with working with children in a group and to easily adapt activities according to their age and the difficulties that were experiencing. Although the intervention was conducted as a group intervention, it had an individualistic approach as the researchers had four postgraduate students in Kinderkinetics that also assisted her in adapting the activities in each lesson and specifically with the smaller children or those that showed challenges with the activities. The program was based on locomotor skills, stationary skills, object manipulation skills, visual–motor skills, grasping as well as vestibular stimulation, reflex inhibition and indirect improvement of physical fitness. This 12-week intervention was presented for 60 min per session and presented twice a week during the morning hours from 10:00 to 11:00. Gross motor skills received the largest amount of attention during the program, because, according to the literature, they are the worst affected if a child is HIV positive. The activities were often alternated and ample rest periods were built in to compensate for the attention span of the children and for fatique. Subsequently a schematic outlay is provided of only the broad outlay of a typical lesson at the beginning and the end of the intervention period to display the progression of the program. It was designed mainly for children in the elementary phase of development as most of the group fell within this phase of development (4–5 years), and the chosen activities were then adapted to be more suitable for children in the initial phase of development (made easier) or to accommodate those that found the activities challenging, while activities were also adapted toward the abilities of older children. The string that was, for example, used for threading was bought from an educational toy store with a hard point that made it easier for smaller children to thread it through holes of which the size was again also adapted according to age. Younger children were also assisted by holding their hands during balancing activities, by decreasing distances or time or amount or making holes bigger. 


**Week 3**

**Week 10**

**Locomotor and physical skills (7 min)**

**Locomotor and physical skills (7 min)**
Crab walking Crab walking through and over different objects (hoops, frisbees) Baboon walking Baboon walking through and over different objects (hoops, frisbees)Frog jumpingFrog jumping over frisbeesHoppingHopping through objectsGallopingGalloping through objects
**Reflexes and Vestibular (5 min)**

**Reflexes and Vestibular (5 min)**
Boat rollingBoat rolling on ball AeroplaneAeroplane on ball Trunk rollsTrunk rolls
**Fine motor (10 min)**

**Fine motor (10 min)**
Pressing sticks into clay and removing them again—using different fingers Attaching laundry pegs to shapes—using different fingers Threading a string through holes in a piece of cardboardThreading string through different forms of noodles
**Locomotor and physical skills (5 min)**

**Locomotor and physical skills (5 min)**
Two-leg jumping Two-leg jumping—over and on small benches Single-leg jumpingOne-leg jumping—around small benchesLearning steps for skipping—hoopSkipping
**Rest period (3 min)**

**Rest period (3 min)**

**Stationary (10 min)**

**Stationary (10 min)**
Standing on 1 leg with eyes open, eyes closedFrisbee—on different body partsWalking on ropes placed in forms—forwards, backwards, oblique, legs crossedStanding on frisbee on one leg with eyes open, eyes closed 
Walking on edge of hoops—forwards, backwards, oblique, legs crossed
**Object manipulation (10 min)**

**Object manipulation (10 min)**
Tossing up a large ball and catching it Tossing up a ball and catching it—bigger children use tennis ballsRolling a ball forward Rolling ball through markersRolling a ball into goal areaKicking a ball softlyKicking a ball Kicking a ball through markersKicking a ball into the goal areaTossing a ball into a bucket—increase distance
**Fine motor (5 min)**

**Fine motor (5 min)**
Coloring a picture Duplicating, cutting out and coloring a picture
**Game (5 min)**

**Game (5 min)**
Parachute game Parachute game with balls

### 2.5. Statistical Analysis

The data were analyzed using Statistica for Windows [34] (StatSoft, Inc S.A., Tulsa, OK, USA 2006). Descriptive statistics were used to determine means (M), standard deviations (SD) and maximum and minimum values. An independent *t*-test was performed to determine whether any statistically significant differences occurred between the HIV-infected and affected children before they were matched into an intervention and control group. Furthermore, a dependent *t*-test was used to compare the differences between the pre-test and post-test results within the groups, as well as within the infected and affected groups. Due to differences found in the pre-test values of the experimental and control group, an ANCOVA was performed to equalize the groups. Adapted means were calculated by means of the ANCOVA, in which the pre-test was used as a co-variable to determine the effect of the intervention based on the post-test means. The level of statistical significance was set at *p* < 0.05. Practical significance of differences (ES) between the testing sessions were calculated by dividing the mean difference (M) between the two testing sessions by the largest standard deviation (SD), as recommended by [35,36] Cohen (1988) and [36] Steyn (1999). Cohen (1988) provides the following cut points to interpret practical significance, namely ES = 0.2 (small effect); ES = 0.5 (medium effect) and ES = 0.8 (large effect). Due to the small number of subjects it was considered practically significant if this effect size indicates medium and larger effects. 

## 3. Results

Table 1 provides information on age and sex characteristics and the number of particiapants included in the study. Table 2 presents the means and standard deviations obtained by the affected and the infected children before they were matched into an intervention and a control group. No statistically significant differences (*p* < 0.05) were found before the onset of the intervention between the infected and affected subjects (Table 2). From this it can be assumed that the groups were more or less similar regarding age, and all the motor skills components measured with the PDMS-2 and the physical skills before they were matched into an intervention and a control group. 

Table 3 displays the descriptive results of the intervention and control groups with regard to the pre-test and the post-tests as well as statistically significant differences between the testing sessions. Table 3 shows that the standard scores of the intervention group improved statistically significantly (*p* < 0.05) in some of the skills during the post-test. These include grasping, the fine motor quotient and percentile, as well as the total motor percentile and quotient (*p* < 0.05). Opposed to this, the control group displayed a statistically significant (*p* < 0.05) deterioration in the fine motor percentile and the fine motor quotient. Lower values were also found in the visual–motor percentile, gross motor percentile and quotient and in the total motor percentile and quotient during the post-test, although these lowering values were not statistically significant (*p* > 0.05). Performance in the remaining motor and physical skills remained more or less at the same level in the control group.

Table 4 displays the results of an ANCOVA to determine the effect of the intervention program. The analysis was performed because t-testing indicated significant intra-group differences during pre-testing between the intervention and control groups; hence these differences had to be adjusted for by means of an ANCOVA (Table 4). These results, where the pre-test was used as a co-variable during the analysis of the data, showed that the motor intervention led to statistically significant improvement in post-test values in the standard score F (1.19) = 4.44 (*p* = 0.04) and the percentile F (1.19) = 5.15 (*p* = 0.03) of locomotor skills, with effect sizes of 0.82 and 0.96, respectively, indicating large practical effects. The percentile F (1.19) = 9.22 (*p* = 0.007) and quotient F (1.19) = 11.396 (*p* = 0.003) of fine motor skills also indicated statistically significant group differences, with effect sizes of 1.66 and 2.02, respectively, which is also indicative of large practical effects. The total motor percentile F (1.19) = 3.81 (*p* = 0.07) and quotient F (1.19) = 4.44 (*p* = 0.05) also improved, with effect sizes again showing large practical effects (0.88 and 0.96).

Table 5 displays the pre-test and post-test results of the infected and affected subjects in the intervention and control groups, respectively, since the second aim of the study was to determine whether infected and affected children would react differently to intervention.The infected children in the intervention group improved statistically significantly regarding their locomotor standard score and percentile as well as their total motor percentile (*p* < 0.05). These improvements also showed large practical effects (ES > 0.8). The affected subjects in the intervention group showed statistically significant (*p* < 0.05) improvement in the fine motor percentile as well as quotient, although the improvements only had small to medium effects (ES > 0.5).

In the control group, the affected subjects deteriorated statistically significantly with regard to the fine motor percentile as well as the quotient (*p* < 0.05), with an ES of 1.27 and 0.17, respectively. Although several values were lower during the post-test in the infected group, no statistically significant changes had occurred in the group.

## 4. Discussion

This study wanted to determine if motor intervention can be beneficial to children infected and affected with HIV and if infected and affected children might react differently to such an intervention. At baseline, the HIV +group’s developmental level was much lower compared to their chronological age (stationary 12 months, grasping 9 months; locomotor skills 11 months, object control skills and visual motor integration 6 months each), and in comparison to an affected group [37]. This agrees with findings indicating that HIV+ children show early onset of motor deficits and are therefore in need of intervention to improve these backlogs as early as possible [2,4,9,11,12,13]. The overall motor, fine motor and the locomotor skills of the children that followed the intervention benefited statistically significantly (*p* < 0.05) from the motor intervention, and these improvements also showed large practical significance (ES > 0.5). The fine motor skills of the intervention group improved statistically significantly (*p* < 0.05) compared to the control group that showed significant deterioration of fine motor skills (*p* < 0.05) during the intervention period. These skills of the intervention group might have benefitted from the activities that improved gross motor functioning, body stabilty and visual–motor coordination, which all play a role in fine motor coordination. Fine motor skills are also considered to be sensitive to rehabilitation and targeted intervention [38]. This is a positive effect of the intervention as it is considered important to improve fine motor skills of children that shows delays in this area before they enter formal schooling, since 50% of a typical school day is spent involving activities that require fine motor activities [39]. In addition, it is also indicated that fast and efficient handwriting have an impact on a child’s scholastic performance [40]. The improvement of locomotor skills in the intervention group can be ascribed to the program been predominantly focused on such skills, seeing that these are the skills which are worst affected, according to the literature [9,16,17,18,19,20,37]. It is also evident from the results that the intervention program improved the overall motor skills repertoire of the intervention group, although not statistically significantly in all the sub skills that contribute to the score of this section. The intervention most probably contributed to improved muscle tone, muscle bulk and muscle strength in the infected children. Literature indicate in this regard that children experiencing such deficits are at an increased risk for disease progression [14]. The largest portion of time during the intervention was, however, devoted to the skills which did improve statistically significantly (locomotor and fine motor skills). Although the overall motor skills of the intervention group also improved statistically significantly, more improvement could possibly have taken place in specific skills, such as stationary (balancing skills) and object control skills, if more time could be spent on the improvement of such skills. Object control skills are considered to be core fundamental motor skills that young children should be exposed to, that can improve perceptual-motor skills but also give them the best possible chance to engage in health-enhancing physical activities. Balancing skills are again important to maintain good posture and can improve muscle tone, which are both found to be compromised in HIV+ children [14]. They should therefore be part of intervention programs that focus on early motor development. Object control skills, however, demand high levels of functional coordination to make controlled contact with implements such as balls or bats and may subsequently need longer exposure to intervention activities for improvement at a young age [41]. Very little change was found in the strength of the treatment group, therefore more specific activities need to be included in a motor intervention of this nature to address strength improvement. The protective effects of strength are, however, important to address in health-enhancing programs as upper- and lower-body strength levels are related to functional limitations, while high muscular strength levels provide protective effects against disability and can improve muscle tone [42]. A weakening of muscles and a drop in energy levels can occur over time in HIV+ children and should be maintained by intervention using especially large muscle groups [37]. This treatment was offered as a group intervention, and it might be that because children differ in the nature but also the extent of their developmental deficits that they might have reacted differently to the intervention. Some might need more exposure to a wide variety of activities, whereby the amount would be the determining factor, while a more qualitative approach might be more important in those skills that are worse affected, which highlights a more individualistic, rather than a group approach during treatment. A possibility is also that the duration of the intervention might have been too short to produce training effects in all these areas.

Our results furthermore showed that the infected children showed more positive improvement as a result of the intervention in locomotor and overall motor skills (*p* < 0.05; ES > 0.8) compared to the affected children in the group. A possible reason could be that the infected children generally had poorer skills during the pre-test with more room for improvement in this group. The responsiveness of these children to motor intervention is also evident from the differences between the pre-test and post-test values which, compared to the infected children in the control group, all indicated larger improvement. The fine motor skills of the affected children in the intervention group also improved statistically significantly, compared to a statistically significant deterioration seen in these skills in the affected children of the control group. As fine motor skils are reported to be responsive to targeted intervention this responsiveness might have added to these positive changes [38]. It therefore seems from the results that children infected and affected by HIV/AIDS can indeed benefit from a motor intervention program, and that infected children are especially receptive to such treatment effects. A few adaptions are consequently recommended to the program content and the duration of the program to improve the outcome. The program was also presented in a group context, and although it is important to promote social inclusion by means of group activities, based on the results attained, it is recommended that as a treatment, a more individualistic approach could be followed for children with HIV/VIGS. Such an approach might lead to more marked improvements in the motor capabilities of each child. Sensory–neurological assessments are also recommended to determine the nature of the underlying problems in order to increase the effect of the intervention.

No similar interventions could, however, be found to compare our results with, although our findings are in agreement with the outcome of a home-based program developed by physiotherapists in South Africa and taught by trained caregivers to improve activities of daily living in 18-month-old HIV+ children [4]. In this study, children in the experimental group showed significantly greater improvement in cognitive (*p* = 0.010) and motor (*p* = 0.020) development over time compared to a control group. Although not comparable with our findings, another complimentary study was performed where massaging was used as a form of immune stimulation on a group of 5 year olds, which also showed promising results [43]. A stimulating program conducted on young children hospitalized with cancer also showed promising results on gains in motor functioning, as reported by their parents [38]. A review on the importance of the physical activity in the care of HIV children furthermore concluded that the negative effects from HIV can be minimized by exposure to the effect of physical activity as it is highly beneficial and safe for children and youth living with HIV, showing positive effects on metabolic, morphological, psychological and functional parameters, as well as self-esteem and self-image, resulting in better quality of life [44] Considering the multidisciplinary characteristic of primary health care, pediatric health practitioners such as kinderkineticists can therefore play a vital part in the team of care by providing complementary treatment effects to the developing child who are challenged by HIV.

A limitation of this study was that the progression of the disease of the children could not be established due to the ethical aspects attached to it. It could possibly have played a role in the execution and improvement of motor skills, because fatique will probably set in at a faster rate in children at more advanced stages of HIV. The dropout of participants from the study after pre-testing, and consequently the small intervention group, also makes it difficult to generalize the findings to a larger population. Although the intervention was applied two times per week, it was only performed over 12 weeks which is shorter than the six months that are recommended to avoid bias due to a learning effect. Despite these limitations, the study does provide novel elements in that it provides indications of how to support children infected with HIV developmentally, in contexts of health care, by means of motor programs.

## 5. Conclusions

This study is distinctive in that it was the first study to use neuro-motor developmental activities as a complimentary treatment in the care of children with HIV or affected by HIV. Although the findings are based on a small number of subjects, and should be considered as exploratory in nature, they provide valuable insight to practitioners and clinicians as an intervention that can address developmental needs in the motor domain that can be used in the management of HIV children. Developmental interventions that stimulate motor development by means of structured motor activities and developmentally appropriate play and games are important to consider to improve the health and wellbeing of these children, who are struggling on a daily basis with the deterioration of their motor skills. Motor skills are considered vital tools of active daily living but also building blocks that are associated with school readiness and school success, which can buffer them against neurocognitive and behavioral problems that can persist and deepen at school age, therefore to optimize their daily lives. Programs of this nature not only appeal to the developmental level of children at a young age, as they brings back a sense of normality and also the fun of being a child into their daily routines while also promoting social inclusion, but they also address developmental shortcomings and potential deterioration in development progress that are associated with HIV risks. It therefore can add to a milieu of stimulation that can be associated with a higher level of general neurocognitive and social development and boosting of health and early growth and development. The outcome of this research is therefore of particular importance as a guide in the planning and implementing of similar studies in the future, and the implementation of such programs is encouraged in the management of young children with HIV. In addition, it can aid caregivers that have to provide supportive care to improve the quality of life of children who live with and are challenged with the realities of this condition. Although caregivers lack clinical expertise, they can be trained to deliver similar, but general motor developmental programs to all children challenged by the disease. Similar studies are also recommended that can improve on the limitations that were found in this study.

## Figures and Tables

**Table 1:** Descriptive characteristics of the experimental and control group.

	Experimental Group (*n* = 9)				Control Group (*n* = 13)				Total	Group
	Boys		Girls		Boys		Girls			
	*N*	Age	*n*	Age	*n*	Age	*n*	Age	*n*	Age
HIV infected	1	46.0	3	50.33	7	53.14	0	-	11	51.73
HIV affected	3	46.0	2	37.5	3	51.67	3	40.33	11	44.45
Total	4		5		10		3		22	

Note: *n* = number of participants; age = mean age in months.

**Table 2:** Differences between the HIV-infected and affected children before matching them into an intervention and control group.

	Infected Participants (*n* = 11)		Affected Participants (*n* = 11)		
	M	SD	M	SD	*p*
Age	51.73	10.15	44.45	10.76	0.1187
Stationary-S	8.18	2.79	9.45	3.05	0.3187
Stationary-P	31.64	25.46	42.64	30.96	0.3736
Locomotor-S	7.45	2.30	9.09	2.74	0.1444
Locomotor-P	25.00	21.48	37.64	26.13	0.2297
Object manip-S	9.45	2.21	9.91	2.43	0.6508
Object manip-P	43.91	25.34	49.00	27.25	0.6549
Grasping-S	9.64	1.96	11.64	3.17	0.0905
Grasping-P	44.82	22.19	64.91	27.15	0.0719
Visual–motor-S	9.45	3.33	8.09	2.26	0.2739
Visual–motor-P	46.18	34.43	30.55	23.52	0.2280
Gross motor-P	30.55	24.29	42.09	25.63	0.2910
Gross motor-Q	89.82	13.48	96.64	11.31	0.2134
Fine motor-P	43.82	25.80	47.64	30.41	0.7541
Fine motor-Q	97.00	11.30	99.18	15.12	0.7056
Total motor-P	34.45	24.35	43.82	28.36	0.4159
Total motor-Q	92.18	12.60	97.18	13.56	0.3810
Handgrip strength-R	4.05	1.68	3.77	2.26	0.7515
Handgrip strength-L	3.27	1.75	3.50	2.10	0.7855
Standing long-jump	42.09	23.12	44.14	34.07	0.8708

Note: M = mean; SD = standard deviation; S = standard score; P = percentile, Q = quotient; R = right; L = left.

**Table 3:** Comparison between the pre-test and the post-test in the intervention and control groups.

	Intervention Group (*n* = 9)					Control Group (*n* = 13)				
	Pre-T		Post-T			Pre-T		Post-T		
	M	SD	M	SD	P	M	SD	M	SD	*p*
Stationary-S	7.44	2.30	8.11	2.85	0.5632	9.77	3.00	9.69	2.72	0.8506
Stationary-P	25.00	17.79	33.44	25.29	0.3718	45.54	31.57	45.08	28.91	0.9172
Locomotor-S	8.22	3.35	10.00	2.40	0.1614	8.31	2.10	8.23	2.35	0.7938
Locomotor-P	29.67	29.73	50.56	25.88	0.0869	32.46	20.84	32.69	22.94	0.9370
Object manip-S	9.44	2.01	10.78	2.22	0.2249	9.85	2.51	9.92	1.93	0.8078
Object manip-P	43.67	22.54	57.33	23.60	0.2419	48.38	28.59	49.00	23.37	0.8449
Grasping-S	9.44	2.19	10.89	2.52	* 0.0499	11.46	2.90	11.15	2.03	0.4874
Grasping-P	44.44	25.27	60.11	26.72	0.0694	62.08	25.41	61.92	21.35	0.9733
Visual–motor-S	7.78	2.77	9.11	2.47	0.2133	9.46	2.82	8.92	2.96	0.3156
Visual–motor-P	28.11	28.23	37.78	24.35	0.3613	45.46	29.94	39.69	31.02	0.3407
Gross motor-P	31.44	23.29	45.22	21.50	0.1965	39.69	26.61	35.62	25.14	0.6412
Gross motor-Q	90.11	13.25	97.67	9.29	0.1757	95.38	12.24	93.62	11.69	0.6508
Fine motor-P	31.67	22.22	51.11	24.73	* 0.0068	55.46	27.42	41.38	31.51	* 0.0432
Fine motor-Q	91.33	10.44	100.00	11.62	* 0.0064	102.77	12.99	94.92	16.81	* 0.0237
Total motor-P	29.67	22.51	47.11	20.70	* 0.0291	45.69	27.44	36.77	28.02	0.2460
Total motor-Q	89.56	11.96	98.22	9.60	* 0.0339	98.23	12.98	93.46	14.12	0.1770
Handgrip strength-R	3.06	2.21	3.32	2.41	0.0941	4.50	1.57	4.65	1.66	0.1654
Handgrip strength-L	3.11	1.95	3.33	1.92	0.1690	3.58	1.90	3.65	2.06	0.5486
Standing long-jump	40.56	28.89	40.78	29.16	0.5588	44.88	29.16	45.81	29.15	0.0821

Note: * *p* < 0.05; M = mean; SD = standard deviation; S = standard score; P = percentile, Q = quotient; Pre-T = Pre-test; Post-T = Post-test.

**Table 4:** Adapted post-test means calculated with an ANCOVA with the pre-test as a co-variable.

	Intervention Group (*n* = 9)		Control Group (*n* = 13)		
	M	SD	M	SD	ES
Stationary-S	8.98	0.78	9.09	0.64	-
Stationary-P	42.22	6.95	39.00	5.71	-
Locomotor-S	10.03	0.66	8.21	0.55	0.82
Locomotor-P	51.53	6.60	32.02	5.49	0.96
Object manip-S	10.89	0.59	9.84	0.49	-
Object manip-P	58.84	6.40	47.96	5.32	-
Grasping-S	11.67	0.51	10.61	0.42	-
Grasping-P	66.94	5.97	57.20	4.91	-
Visual–motor-S	9.74	0.75	8.49	0.62	-
Visual–motor-P	44.05	7.81	35.35	6.44	-
Gross motor-P	46.34	7.94	34.84	6.59	-
Gross motor-Q	98.35	3.61	93.14	3.00	-
Fine motor-P	62.84	7.19	33.26	5.87	1.66
Fine motor-Q	106.45	3.50	90.46	2.85	2.02
Total motor-P	52.35	7.41	33.14	6.11	0.88
Total motor-Q	101.25	3.57	91.36	2.94	0.95
Handgrip strength-R	4.22	0.14	4.03	0.11	-
Handgrip strength-L	3.61	0.15	3.46	0.13	-
Standing long-jump	43.34	0.52	44.03	0.43	-

Note: M = mean; SD = standard deviation; ES = effect size; S = standard score; P = percentile; Q = quotient.

**Table 5:** Comparison of the pre- and post-test results of the infected and affected children in the intervention and control groups.

Intervention Group	Infected Group (*n* = 4)							Affected Group (*n* = 5)						
	Pre-T		Post-T					Pre-T		Post-T				
	M	SD	M	SD	Diff	p	ES	M	SD	M	SD	Diff	*p*	ES
Stationary-S	7.25	3.1	8	2.58	0.75	0.689	-	7.6	1.82	8.2	3.35	0.6	0.73	-
Stationary-P	26	21.8	30.3	25.6	4.25	0.751	-	24.2	16.6	36	27.8	11.8	0.44	-
Locomotor-S	6.5	1.73	9.75	1.89	3.25	* 0.022	1.72	9.6	3.85	10.2	2.95	0.6	0.77	-
Locomotor-P	15	14.8	48	22.2	33	* 0.038	1.49	41.4	34.9	52.6	31	11.2	0.57	-
Object manip-S	8.25	1.71	11.5	2.65	3.25	0.184	-	10.4	1.82	10.2	1.92	−0.2	0.70	-
Object manip-P	30.3	17.5	64.3	25.9	34	0.182	-	54.4	21.5	51.8	22.9	−2.6	0.70	-
Grasping-S	8.25	0.96	10.8	1.5	2.5	0.096	-	10.4	2.51	11	3.32	0.6	0.37	-
Grasping-P	28.8	10.2	59.3	19	30.5	0.09	-	57	27.5	60.8	34	3.8	0.53	-
Visual–motor-S	7.75	3.59	8.5	0.58	0.75	0.729	-	7.8	2.39	9.6	3.36	1.8	0.15	-
Visual–motor-P	28.5	37.3	31	6.93	2.5	0.912	-	27.8	23.5	43.2	32.7	15.4	0.16	-
Gross motor-P	21.5	18.9	46.3	11.7	24.8	0.069	-	39.4	25.3	44.4	28.6	5	0.77	-
Gross motor-Q	84	14.7	98.5	4.43	14.5	0.147	-	95	11.1	97	12.5	2	0.77	-
Fine motor-P	21.5	13.8	44.3	13.2	22.8	0.119	-	39.8	25.7	56.6	31.7	16.8	* 0.05	0.53
Fine motor-Q	87.3	6.65	97.8	5.12	10.5	0.11	-	94.6	12.4	102	15.5	7.2	* 0.04	0.46
Total motor-P	18.5	14.8	44.3	10.1	25.8	* 0.046	1.77	38.6	25	49.4	27.7	10.8	0.33	-
Total motor-Q	84	10.8	97.8	3.86	13.8	0.079	-	94	12	98.6	13.2	4.6	0.31	-
Handgrip strength-R	2.38	0.48	2.75	0.29	0.37	0.215	-	3.6	2.97	3.78	3.31	0.18	0.37	-
Handgrip strength-L	2.63	0.48	2.88	0.25	0.25	0.391	-	3.5	2.65	3.7	2.64	0.2	0.37	-
Standing long-jump	39.3	34	39.3	34	0		-	41.6	28.3	42	28.8	0.4	0.59	-
**Control Group**	**Infected Group (*n* = 7)**							**Affected Group (*n* = 6)**						
Stationary-S	8.71	2.69	9.14	3.02	0.43	0.53	-	11	3.1	10.3	2.42	−0.7	0.10	-
Stationary-P	34.9	28.5	38.6	30.4	3.71	0.64	-	58	32.8	52.7	27.8	−5.3	0.12	-
Locomotor-S	8	2.52	8.14	2.79	0.14	0.77	-	8.67	1.63	8.33	1.97	−0.3	0.36	-
Locomotor-P	30.7	23.6	33.3	26.5	2.58	0.58	-	34.5	19.2	32	20.5	−2.5	0.50	-
Object manip-S	10.1	2.27	9.86	1.95	−0.3	0.17	-	9.5	2.95	10	2.1	0.5	0.47	-
Object manip-P	51.7	26.9	48.7	23.7	−3	0.18	-	44.5	32.6	49.3	25.3	4.83	0.47	-
Grasping-S	10.4	1.99	10.6	1.27	0.14	0.84	-	12.7	3.5	11.8	2.64	−0.8	0.14	-
Grasping-P	54	22.3	56.7	15	2.71	0.76	-	71.5	27.5	68	27.3	−3.5	0.15	-
Visual–motor-S	10.4	2.99	9.57	3.05	−0.9	0.37	-	8.33	2.34	8.17	2.93	−0.2	0.74	-
Visual–motor-P	56.3	30.9	46.7	32.2	−9.6	0.38	-	32.8	25.5	31.5	30.3	−1.3	0.80	-
Gross motor-P	35.7	26.8	30.3	27	−5.4	0.74	-	44.3	28.1	41.8	23.6	−2.5	0.64	-
Gross motor-Q	93.1	12.6	90.7	13	−2.4	0.75	-	98	12.4	97	9.94	−1	0.65	-
Fine motor-P	56.6	22.2	34.7	30.3	−22	0.09	-	54.2	34.8	49.2	33.9	−5	* 0.06	1.27
Fine motor-Q	103	9.55	90.6	17.3	−12	0.06	-	103	17.2	100	16.2	−3	* 0.04	0.17
Total motor-P	43.6	24.7	30	28.4	−14	0.35	-	48.2	32.6	44.7	27.9	−3.5	0.40	-
Total motor-Q	96.9	11.7	89.4	14.9	−7.4	0.27	-	99.8	15.3	98.2	12.7	−1.7	0.36	-
Handgrip strength-R	5	1.29	5.14	1.35	0.14	0.36	-	3.92	1.77	4.08	1.93	0.16	0.36	-
Handgrip strength-L	3.64	2.14	3.71	2.45	0.07	0.77	-	3.5	1.79	3.58	1.72	0.08	0.36	-
Standing long-jump	43.7	17.5	44.3	17.2	0.58	0.17	-	46.3	40.9	47.6	41	1.33	0.24	-

Note: * *p* < 0.05; M = mean; SD = standard deviation; ES = effect size; S = standard score; P = percentile; Q = quotient; Pre-T = Pre-test; Post-T = Post-test; Diff = difference between Pre-T and Pos-T.

## Data Availability

Not applicable.
